# Editorial: Mindfulness and mental health in the time of the COVID-19 pandemic

**DOI:** 10.3389/fpsyg.2023.1209911

**Published:** 2023-05-10

**Authors:** Wendy Wen Li, Daniel Miller, Shauna Shapiro, Annalakshmi Narayanan

**Affiliations:** ^1^Department of Psychology, James Cook University, Townsville, QLD, Australia; ^2^School of Education and Counseling Psychology, Santa Clara University, Santa Clara, CA, United States; ^3^Department of Psychology, Bharathiar University, Coimbatore, India

**Keywords:** mindfulness, mental health, COVID-19, mindfulness-based intervention, anxiety

COVID-19, associated public health measures—such as quarantine lockdown, the cessation of lockdown, re-lockdown—and media coverage of the spread of the virus, have contributed to psychological fear and anxiety on a global scale. As of 6 April 2023, globally 762,201,169 confirmed cases of COVID-19 and 6,893,190 deaths have been reported to WHO. Many people have been able to effectively engage mental resources, such as mindfulness, to overcome pandemic-related adversities and stresses and maintain mental health. This Research Topic, including seven papers, aims to explore the impact of mindfulness on the maintenance (and even potential improvement) of psychological wellbeing and mental health in the face of the COVID-19 pandemic.

Mindfulness refers to the quality of being aware of the present moment with curious, open-minded, non-judging, non-striving, and acceptant attitudes (Kabat-Zinn, 1982, 2013; Shapiro et al., 2008; Fisher et al., 2023). Mindfulness can provide cognitive flexibility to support the individual to actively construct current experiences and adapt to changing demands and challenging situations brought about by the COVID-19 pandemic by building mental resources and establishing new perspectives (Li et al., 2020).

Research indicates that greater mindfulness is associated with less mental distress in the time of the COVID-19 pandemic. In this Research Topic, Xu et al.'s systematic review and meta-analysis of 12 correlational studies show that mindfulness negatively correlated with anxiety and depression. In their cross-sectional study, Zakeri et al. reported that mindfulness negatively correlated with secondary traumatic stress and burnout among nurses. Zakeri et al. also found that mindfulness positively correlated with psychological hardiness; the ability to cope with stressful situations through focusing on human inner experience and mental perception that regard challenges as opportunity for growth. Greater mindfulness was associated with higher level of psychological hardiness which in turn was associated with less mental distress. The mediating effect of psychological hardiness on the relationship between mindfulness and mental distress warrants future studies.

Mindfulness-based interventions have been employed to support people to maintain mental health during the COVID-19 pandemic. Three papers in this Research Topic provide empirical evidence on the effectiveness of mindfulness-based interventions for improving mental health in such a difficult time. Online interventions gained popularity during the pandemic as a result of lockdown measures. In their randomized controlled trial (RCTs) of a 17-day online mindfulness meditation intervention for university students, Devillers-Réolon et al. found that people in the intervention group, compared to their counterparts in the control group, showed lower levels of stress, anxiety and depression, and higher levels in subjective wellbeing. The results of a single-arm trial of a 2-month workplace mindfulness intervention (delivered in person, online, and in a hybrid format due to the changing COVID-19 situation) for information communications technology (ICT) managers suggested significant improvement in wellbeing (WHO-5) at post-test and follow-up test (Schubin et al.). The findings from another single-arm trial of a 12-week online mind-body based intervention for nurses reported significant increases in mindfulness and reductions in negative emotions and stress at 6 months follow-up. However, the sample also displayed reduced subjective wellbeing and resilience at follow-up (Cepeda-Lopez et al.). Considering limitations of single-arm trials in confirming the efficacy of interventions, the findings of Cepeda-Lopez et al. and Schubin et al. should be generalized with caution.

From a mindfulness practice perspective, Micheli et al. and Thompson argued that greater mindfulness practice is associated with higher levels of emotional regulation, self-efficacy and resilience. Micheli et al. found that advanced mindfulness practitioners reported significantly higher levels of adaptive emotion regulation compared to non-practitioners. Reflecting on his personal journey of mindfulness practice, Thompson suggested that mindfulness practice promotes self-efficacy and resilience, which in turn contributes to psychological wellbeing and mental health.

In summary, this Research Topic sheds light on the relationship between mindfulness and mental health; that is, mindfulness may act as a protective factor for maintaining good mental health during the COVID-19 pandemic. Including mindfulness in post-COVID-19 pandemic mental health interventions and in prevention programs for future pandemics may help attenuate the effects of the COVID-19 and future public health crises on mental ill health.

## Author contributions

WL substantially contributed to drafting and DM substantially contributed to critically reviewing the editorial. All authors have made substantial contributions to the concept of the editorial. All authors contributed to the article and approved the submitted version.
